# Molecularly Imprinted Polymer Nanoparticles: An Emerging Versatile Platform for Cancer Therapy

**DOI:** 10.1002/anie.202005309

**Published:** 2020-11-23

**Authors:** Shuxin Xu, Lisheng Wang, Zhen Liu

**Affiliations:** ^1^ State Key Laboratory of Analytical Chemistry for Life Science School of Chemistry and Chemical Engineering Nanjing University 163 Xianlin Avenue Nanjing 210023 China; ^2^ Department of Biochemistry, Microbiology and Immunology Faculty of Medicine University of Ottawa 451 Smyth Road Ottawa Ontario K1H 8M5 Canada

**Keywords:** cancer therapy, drug delivery, molecular imprinting, nanoparticles

## Abstract

Molecularly imprinted polymers (MIPs) are chemically synthesized affinity materials with tailor‐made binding cavities complementary to the template molecules in shape, size, and functionality. Recently, engineering MIP‐based nanomedicines to improve cancer therapy has become a rapidly growing field and future research direction. Because of the unique properties and functions of MIPs, MIP‐based nanoparticles (nanoMIPs) are not only alternatives to current nanomaterials for cancer therapy, but also hold the potential to fill gaps associated with biological ligand‐based nanomedicines, such as immunogenicity, stability, applicability, and economic viability. Here, we survey recent advances in the design and fabrication of nanoMIPs for cancer therapy and highlight their distinct features. In addition, how to use these features to achieve desired performance, including extended circulation, active targeting, controlled drug release and anti‐tumor efficacy, is discussed and summarized. We expect that this minireview will inspire more advanced studies in MIP‐based nanomedicines for cancer therapy.

## Graphical abstract

This minireview surveys recent advances in research on molecularly imprinted polymer nanoparticles (nanoMIPs) for cancer therapy and highlights the distinct features of nanoMIPs for the rational design and construction of cancer nanomedicines with desired performances, including extended circulation, active targeting, controlled drug release, and antitumor efficacy.

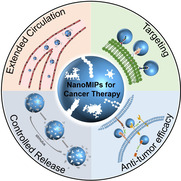

## 1 Introduction

Cancer is a major public health problem and has been one of the leading causes of death worldwide.^[1]^ Nanomedicines based on polymers, liposomes, inorganic particles, carbon materials, metallic materials, and their composites have emerged as promising platforms for the diagnosis and treatment of malignant tumors over the past decades, contributing to decreased mortality and prolonged lifetime.^[2]^ The elaborate design of nanomedicines for cancer therapy is expected to overcome a series of biological barriers and pitfalls, improve the pharmacokinetic and pharmacodynamic profiles of conventional therapeutics, and optimize the therapeutic efficacy with minimal side effects.^[3]^ The advances in smart nanomaterials are one of the driving forces for the development of cancer nanomedicine.^[4]^

Molecularly imprinted polymers (MIPs), also referred to as plastic antibodies or artificial antibodies, are chemically synthesized affinity materials with tailor‐made binding cavities complementary to the template molecules in shape, size, and functionality.^[5]^ Owing to the presence of imprinted cavities, MIPs can specifically recognize and bind the template molecules. Compared to traditional bioligands such as antibodies, aptamers, and lectins, MIPs feature several advantages, such as easy preparation, excellent stability, and low cost.^[6]^ In recent years, significant progress in the performance of MIPs including specificity, affinity, and targeting scope has motivated researchers to expand their traditional application fields, such as separation, sensing, and assays, to more challenging biomedical applications, such as disease diagnosis, bioimaging, and cancer targeting.^[7]^

MIP‐based nanoparticles (nanoMIPs) have attracted great attention due to their easy combination of the biomimetic recognition of MIPs with the unique properties of nanoparticles (nanoscale size, high surface‐to‐volume ratio, and optical, acoustic, thermal, magnetic, and electric properties).^[8]^ Particularly, as a new platform in cancer nanomedicine, nanoMIPs have exhibited great potential for cancer therapy.^[9]^ The unique features of nanoMIPs bring versatile and desired functions to cancer nanomedicine. Moreover, the realization of these properties and functions is independent of bioligands. Thus, nanoMIPs provide not only an alternative to the currently existing nanomaterials for cancer therapy, but also hold great potential to fill the gaps in nanomedicines associated with the dependence on biological ligands, for example immunogenicity, stability, applicability, and cost.

Recently, several excellent reviews on the fundamental chemical design, preparation strategies, and biomedical applications of nanoMIPs (e.g. bioimaging, disease diagnosis, and drug delivery) have been published.^[10]^ MIPs as synthetic antibodies for cancer therapy have also been highlighted.^[11]^ However, an overview specifically focused on the recent advances in nanoMIPs for cancer therapy is apparently lacking. To fill this gap, in this minireview, we survey recent advances in the rational design of MIP‐based nanomedicines for cancer therapy. Specifically, we highlight the distinct features of MIPs that have been used to rationally design and construct cancer nanomedicines with desired performance. For cancer therapy, intravenously administered nanomedicines must evade the reticuloendothelial system during systemic circulation, permeate the tumor site via tumor vessel leakage, penetrate deep into the tumor tissue, be internalized by tumor cells, and exert a therapeutic effect on predesigned targets.^[3]^ Along these lines, we discuss the functions of nanoMIPs for cancer therapy in four aspects: extended circulation, active targeting, controlled drug release, and antitumor efficacy. In addition, we further introduce some nanoMIPs with dual functions. Finally, we briefly discuss the present challenges and future perspectives of nanoMIPs for cancer therapy.

## 2 Extended Circulation

Defective tumor vessels and impaired lymphatic drainage allow intravenously delivered nanomedicines to preferentially accumulate in tumors, which is termed the passive targeting of cancer nanomedicine.^[12]^ Sufficiently prolonged circulation contributes to the increased accumulation of nanomedicines in tumors. Therefore, rationally designed nanomedicines avoiding rapid clearance in vivo are essential for the efficiency of passive targeting. Poly(ethylene glycol) is the most widely employed polymer for the surface modification of nanoparticles, which can delay phagocytic clearance and extend circulation time by hindering the adsorption of opsonic proteins.^[13]^ Another biologically inspired strategy to extend the circulation time of nanomedicine emerged recently. Nanoparticles can be disguised by cell membrane ligands or bioligands as autogenous cells to escape immune system recognition and elimination, thus prolonging the circulation time in the blood.^[14]^ However, the modification of nanoparticles with ligands complicates their preparation and alters their surface properties and stability.

**Figure 1:**
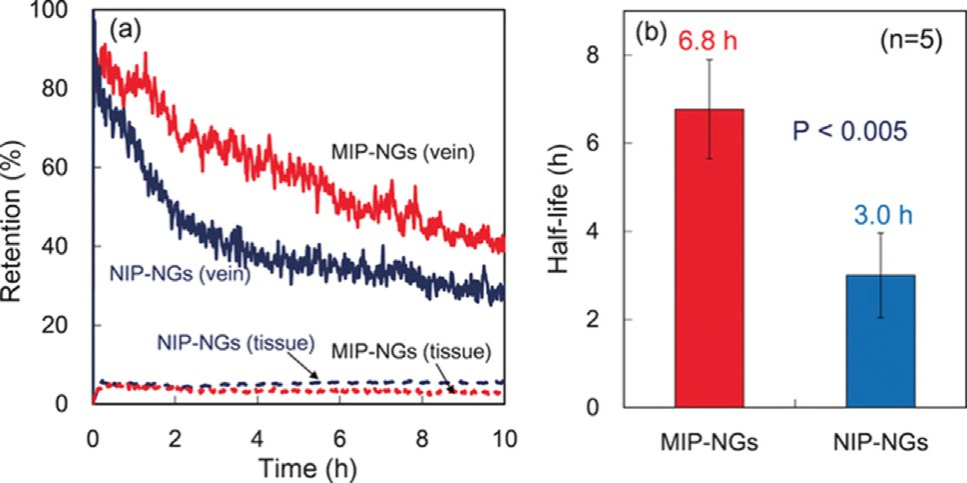
a) Retention and b) half‐life of HSA‐imprinted nanogels (MIP‐NGs) and non‐molecularly imprinted nanogels (NIP‐NGs) in the blood stream (*n*=5). P values were calculated from a two‐tailed t‐test.^[15]^ Reprinted with permission. Copyright 2017, Wiley‐VCH.

Inspired by the biomimetic principle, Toshifumi Takeuchi et al. engineered and prepared molecularly imprinted nanogels by emulsifier‐free precipitation polymerization with human serum albumin (HSA) as an imprinting template and pyrrolidyl acrylate, isopropyl acrylamide, methacryloyloxyethyl phosphorylcholine as functional monomers.^[15]^ The enhanced binding activity and high selectivity of the HSA‐imprinted nanogels to HSA was confirmed with a surface plasmon resonance (SPR) sensor. After intravenous injection into mice, the HSA‐imprinted nanogels interacted with HSA in blood, inducing the formation of an albumin‐rich protein corona around the nanogels. An in vivo imaging experiment demonstrated that HSA‐imprinted nanogels acquired stealth capability with a longer retention time in blood vessels (Figure 1) and little accumulation in liver. Importantly, HSA‐imprinted nanogels exhibited a time‐dependent and approximately sixfold higher accumulation in tumors than in normal organ regions. Without extra modification and with just “self” makers, the nanoMIPs can use the intrinsic biomolecules to camouflage themselves to inhibit phagocytosis, leading to extended circulation and tumor accumulation.^[15]^ Following this concept, more new nanoMIPs can be expected and used for passive targeting in cancer nanomedicine.

## 3 Active Targeting of Tumor Cells

Malignant cells co‐exist with non‐malignant stromal cells including fibroblasts, phagocytes, endothelial cells, and perivascular cells embedded in a protein‐rich extracellular matrix and interstitial fluid, all of which constitute a complex tumor microenvironment.^[16]^ After extravasation of nanomedicines from the systemic circulation to the tumor sites, selective targeting of cancer cells is crucial. The specifically or highly expressed biomolecules in cancer cells related to tumor growth, proliferation, angiogenesis, and metastasis serve as potent candidates for tumor targeting.^[17]^ Various nanomedicines for active targeting have been developed to improve tumor localization and increase tumor retention.[^3b^, ^18^] The active targeting of nanomedicines usually relies on biological ligands including antibodies, aptamers, and peptides.^[19]^ However, there are a number of unresolved issues for the use of biological ligands. The screening and preparation of biological ligands with high quality to target biomolecules is costly, time‐consuming, and sometimes impossible. In vivo instability and the inherent immunogenicity of biological ligands are other challenges in cancer nanomedicine. In contrast, without modification of biological ligands, nanoMIPs exhibit active targeting owing to their intrinsic specific recognition. Thus, nanoMIPs provide potential alternatives to existing biological‐ligand‐based nanomedicines for active targeting.

### 3.1 Targeting Receptor Proteins

Specific surface receptor proteins overexpressed by tumor cells provide ideal targets for the recognition of tumor cells. Targeted therapies by targeting receptor proteins overexpressed on tumor cell surface (e.g. integrin or CD20) enhance tumor cell killing and/or reduce off‐target effects, and some of these have been successfully translated to clinical cancer therapy such as Trastuzumab, Pertuzumab, and Ibritumomab.^[20]^ Receptor protein‐binding MIPs have been exploited for the development of active targeting nanomedicines. The reported nanoMIPs that are able to recognize surface receptor proteins expressed on cancer cells as well as their performance are summarized in Table 1. The dissociation constant (*K*_d_) is a parameter representing the affinity of a MIP towards the target molecule. Selectivity represents the specificity of a MIP to the target molecule, which is often quantitatively described by the cross‐reactivity towards nontarget compounds.

**Table 1:** NanoMIPs recognizing the surface receptor proteins of cancer cells for cancer therapy.

Target	Recognition site	*K*_d_	Selectivity	Performance	Ref.
CA125	whole protein	–	cross‐reactivity was ca. 22 %, 9 %, and 9 % for histidine, immunoglobulin, and glucose	increase cytotoxicity of the loaded drug against target cancer cells; enhance cellular uptake	[21]
					
VEGF	linear peptide epitope	1.6 nM to template epitope	low binding to nontarget vancomycin and peptide epitope of epidermal growth factor receptors	specifically target VEGF over‐expressing melanoma cells xenografted in zebrafish embryos.	[9a]
					
EGFR	linear peptide epitope	7.7 nM to template epitope; 3.6 nM to the extracellular domain of target protein	–	selectively recognize tumor cells over‐expressing EGFR; enhance cytotoxicity of the loaded drug against cancer cells	[9b]
					
CD59	linear peptide epitope	–	–	increase cellular uptake of tumor cells highly expressing CD59 protein; enhance antitumor efficacy of the loaded drugs and photosensitizers in vivo	[27c]
					
HER2	linear peptide epitope	ca.1.1 mM to template epitope^[a]^	–	specifically target HER2‐positive cancer cells	[27b]
	conformational peptide epitope	–	–	enhance antitumor efficacy and distribution of the loaded drugs at tumor site; improve survival rate in a subcutaneous mouse tumor model	[30]
					
P32	conformational peptide epitope	340 nM to target protein	no specific binding to phospholipase A2 (possesses an N‐terminal α‐helix) or mouse nerve growth factor	improve cellular uptake by p32‐positive cancer cells; enhance accumulation at tumor site in a subcutaneous mouse model implanted with p32‐positive cancer cells and intracranially implanted breast cancer cells; improve efficacy of photodynamic treatment in a subcutaneous mouse tumor model	[9c]
	conformational peptide epitope	–	–	promote agent‐phagocytosis by p32‐ overexpressing cancer cells; improve antitumor efficacy of the loaded drug in a subcutaneous mouse tumor model	[28]
					
FN14	conformational peptide epitope	–	no specific binding to scrambled peptide	enhance cellular association and endocytosis by FN14‐positive cells; highly accumulate at tumor site in a subcutaneous mouse tumor model; enhance tumor penetration	[29]
	linear peptide epitope	–	mismatched peptide imprinted polymers exhibited 43.89 % or 32.98 % adsorption amount of the target peptide imprinted polymers to target peptide	target FN‐positive cancer cells; promote cellular uptake; improve antitumor efficacy of the loaded drugs in a subcutaneous mouse tumor model	[27a]
					
Claudin‐4	conformational peptide epitope	–	–	enhance cellular uptake by Claudin‐4‐positive cancer cells	[29]
					
FR‐α	linear peptide epitope	28.1 μM to Hela cells expressing folate receptor‐α	no specific binding to P32 protein or FN14 protein.	target FR‐α over‐expressing cancer cells and enhance cellular uptake; highly accumulate at tumor site in a subcutaneous mouse tumor model	[9d]

[a] Calculated from the original data in the research articles.

Using entire protein‐templated MIPs is a direct strategy to construct active targeting nanomedicines. As an example, cancer‐specific antigen 125 (CA 125) was used as a template and imprinted on the surface of graphene oxide with dopamine as a monomer.^[21]^ The CA 125‐imprinted graphene oxide showed specific binding capacity to CA 125 with an imprinting factor (IF) value of 6.4. IF is defined as the ratio of saturated adsorption of the template on a prepared MIP over a non‐imprinted polymer (NIP) prepared with the same imprinting procedure in the absence of template, which reflects the binding capability of the MIP over the NIP. When the antitumor drug doxorubicin (DOX) was loaded, the CA 125‐imprinted graphene oxide led to the increased drug uptake by tumor cells overexpressing CA 125, and enhanced cytotoxicity against tumor cells. However, to avoid the conformational change of proteins, aqueous or biocompatible polymerization conditions are required for the preparation of entire protein‐imprinted polymers, which becomes a challenge and a limitation. In addition, due to their large molecular sizes, proteins are often difficult to remove from highly cross‐linked polymer networks. Moreover, as the imprinted cavities are associated with a large surface decorated with a wide variety of functional groups, entire protein‐imprinted MIPs are inclined to cause higher cross‐reactivity.^[22]^ Therefore, entire protein‐imprinted MIPs for active targeting have been limited in the field of cancer nanomedicine.

To circumvent the limitations of entire protein‐templated MIPs in recognizing receptor proteins, epitope‐imprinted polymers have been developed to recognize receptor proteins on tumor cells. Epitope imprinting was initially proposed by Rachkov and Minoura^[23]^ and further developed by Shea and co‐workers.^[24]^ Although only a short peptide fragment (epitope) that can represent whole proteins or peptides was utilized as the imprinting template, the synthesized MIPs can recognize whole proteins or peptides carrying the epitope peptide.^[25]^ Compared with entire protein‐templated MIPs, the epitope‐imprinted polymers feature significant advantages with improved affinity to target proteins, reduced nonspecific binding, easy template synthesis, and widely applicable synthesis conditions.^[26]^ These features make epitope‐imprinted polymers highly desirable for whole proteins/peptides that are stable only under physiological conditions or rarely available. The epitope imprinting techniques have been thoroughly reviewed.^[7e]^ Based on their merits, epitope‐imprinted polymers are now widely adopted for developing nanoMIPs in cancer therapy.

**Figure 2:**
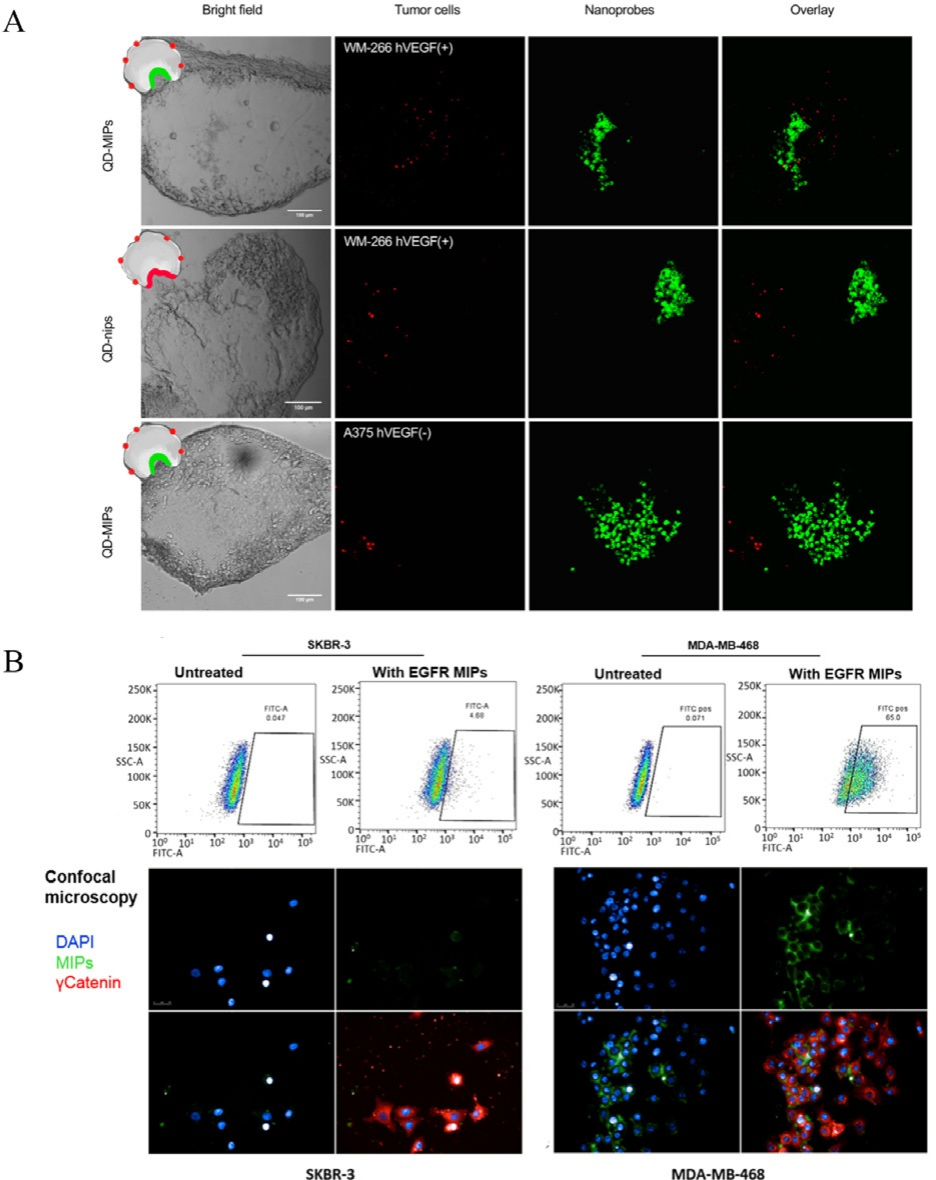
A) Images of human melanoma cells (green) and the fluorescent hVEGF epitope‐imprinted nanoprobes (red), demonstrating the ability of hVEGF epitope‐imprinted nanoprobes to localize cancer cells overexpressing hVEGF in zebrafish embryos.^[9a]^ Reprinted with permission. Copyright 2017, American Chemical Society. B) FACS analysis and confocal microscopy of binding specificity for fluorescent EGFR epitope‐imprinted nanoparticles to two breast cancer cell lines expressing different levels of EGFR. SKBR‐3 cells express low amounts of EGFR while MDA‐MB‐468 cells express high amounts of EGFR, confirming the specific binding of EGFR epitope‐imprinted nanoparticles (green) to the target protein.^[9b]^ Reprinted with permission. Copyright 2018, American Chemical Society.

A linear peptide epitope of vascular endothelial growth factor (VEGF) was used as an imprinting template to prepare nanoMIP against human VEGF.^[9a]^ The nanoMIP was further coupled with quantum dots (QDs) to generate a fluorescent hybrid nanoprobe, named QD‐MIP. The QD‐MIP exhibited excellent affinity for the epitope of VEGF with *K*_d_ in the nanomolar range (1.6 nM), while showing low binding to nontarget molecules.^[9a]^ The binding ability of the QD‐MIP to the whole human VEGF was also demonstrated. Though no concrete imprinting parameter on the protein level was provided, the QD‐MIP specifically targeted VEGF‐overexpressed human melanoma cells xenografted in zebrafish embryos (Figure 2 A). In another study, using a linear peptide epitope of epidermal growth factor receptors (EGFR) as the imprinting template, epitope‐imprinted nanoMIP was prepared through a solid‐phase imprinting strategy.^[9b]^ The nanoMIP exhibited high affinity, with a *K*_d_ of 7.7 nM for binding with the peptide and a *K*_d_ of 3.6 nM for binding with the extracellular domain of EGFR protein.^[9b]^ Consequently, the nanoMIP was capable of selectively recognizing tumor cells overexpressing EGFR, which was demonstrated by flow cytometric analysis and confocal fluorescence imaging (Figure 2 B). Similarly, the linear peptide epitopes of human fibroblast growth factor‐inducible 14 (FN14), human epidermal growth factor receptor 2 (HER2), and CD59 were used as imprinting templates to prepare nanoMIPs for specifically binding receptor proteins overexpressed in different solid tumors.^[27]^

Since some peptide epitopes exhibit a specific and spatial conformation when integrated in the entire protein, linear epitope imprinted polymers, especially when applied in biological systems, can hardly recognize a target epitope with a specific conformation. Therefore, conformational peptide epitope‐imprinted polymers have emerged as a potent compliment for linear peptide epitope‐imprinted polymers in cancer nanomedicine.

**Figure 3:**
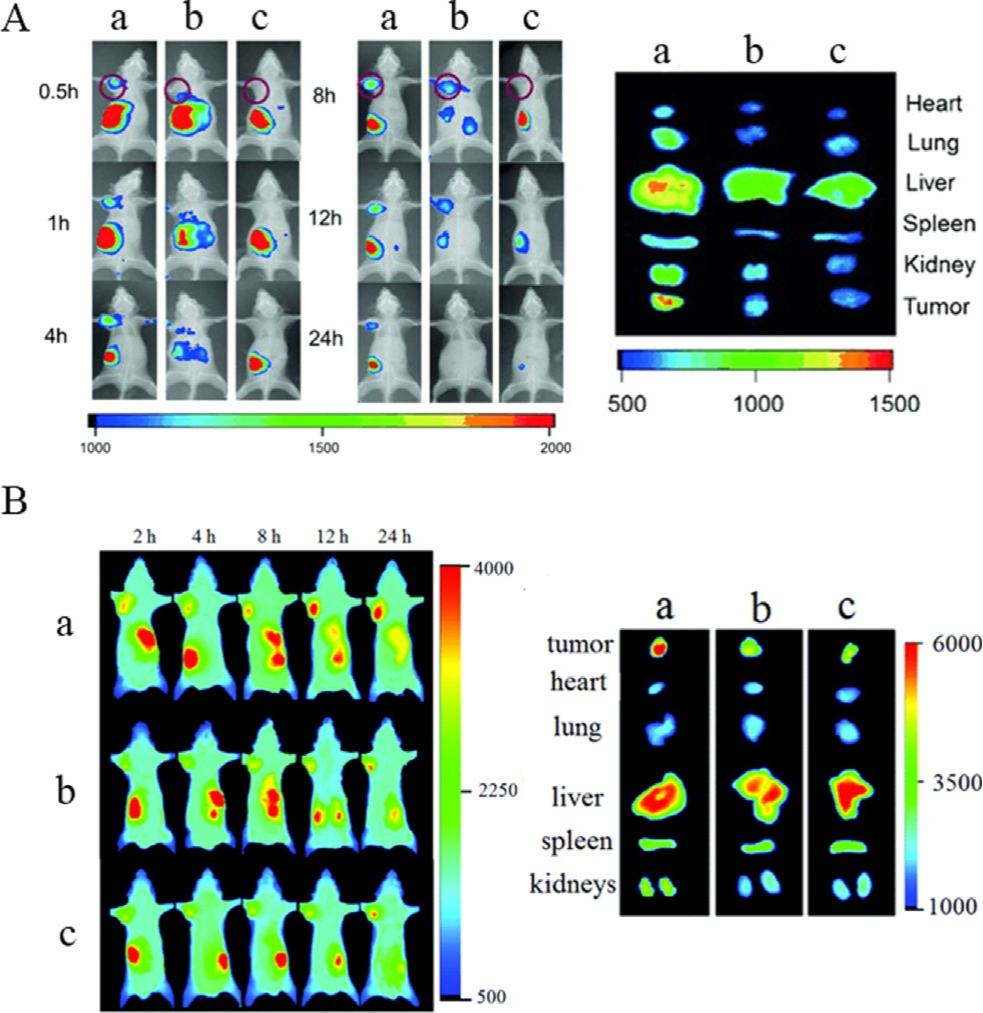
In vivo nanoparticle distribution in a subcutaneous tumor‐bearing mice model at different time points and fluorescence images of major organs and tumors ex vivo at 24 h post‐injection. A) a. Conformational epitope of p32‐imprinted nanoparticles, b. conformational epitope of Lyp‐1 (a peptide ligand binding to the N‐terminal domain of p32) imprinted nanoparticles, c. non‐imprinted nanoparticles.^[9c]^ Reprinted with permission. Copyright 2015, Wiley‐VCH. B) a. Conformational epitope of FRα‐imprinted nanoparticles, b. scrambled epitope of FRα‐imprinted nanoparticles, c. non‐imprinted nanoparticles.^[9d]^ Reprinted with permission. Copyright 2017, Royal Society of Chemistry.

Membrane protein p32 is overexpressed on the surface of a variety of tumor cells. The N‐terminal with α‐helix structure serves as a binding site for p32 recognition. To recognize the N‐terminal with α‐helix structure, a novel polypeptide HAPPE was used as an imprinting template to prepare conformational epitope‐templated nanoMIP.^[9c]^ Through polymerization in deionized water and trifluoroethanol, the α‐helix conformation of the peptide was preserved during the imprinting process. The resulted nanoMIP was capable of specifically binding the conformational epitopes as well as the linear epitopes. Importantly, the synthesized nanoMIP was capable of specifically binding recombinant p32 with a *K*_d_ of 340 nM, leading to greater cellular uptake by p32‐positive cancer cells than with control NIP nanoparticles. As a result, the nanoMIP exhibited enhanced accumulation in p32‐positive tumors in a mouse model inoculated with 4T1 breast cancer cells (Figure 3 A). Combined with photodynamic treatment, the nanoMIP potently inhibited tumor growth in a subcutaneous mouse tumor model. By comparison, another nanoMIP using a linear analogue of HAPPE as the imprinting template exhibited poor uptake by p32‐positive cancer cells and low accumulation in tumors.^[9c]^ The targeting capability of a nanoMIP in vitro and in vivo was also reported where an N‐terminal epitope of p32 with α‐helix structure was used as an imprinting template.^[28]^

In other interesting research, an N‐terminal peptide epitope of folate receptor‐α (FR‐α) was forced to fold into an α‐helix structure as an imprinting template. The synthesized nanoMIP could bind the template peptide and change the target peptide from a disordered to an ordered conformation, thus “creating” binding sites in target receptors.^[9d]^ The nanoMIP specifically targeted FRα‐overexpressing HeLa cells without being affected by the natural ligand, folate, in vitro and in vivo (Figure 3 B). Apart from the extracellular domain, the transmembrane domain with the α‐helix structure of FN14 was also chosen as the imprinting template to synthesize nanoMIP.^[29]^ The prepared nanoMIP exhibited specific binding to template peptide, which induced greater cell endocytosis and superior tumor penetration capability in FN14‐positive tumors.

In addition, conformational epitopes can also be designed by in silico methods according to the protein sequence. Hamid et al. calculated the epitopes of HER2, then selected and synthesized the most powerful epitope.^[30]^ The imprinting was conducted on the surface of silica nanoparticles with the conformational epitope of HER2 and DOX as co‐templates and dopamine as the monomer. Though the specific targeting ability to HER2 was not validated at the molecular or cellular level in an subcutaneous tumor model, the synthesized nanoMIP could specifically target ovarian cancer, as evidenced by the higher DOX concentration in the tumor tissues, enhanced antitumor efficacy, and prolonged survival of mice.

Various nanoMIPs have been successfully constructed for the active targeting of tumor cells by recognizing the tumor‐associated receptor proteins with encouraging results.[^9a^, ^9b^, ^9c^, ^9d^, ^27^, ^28^, ^29^, ^30^] However, several challenges still remain in this direction. Entire protein‐imprinted polymers are difficult to prepare, especially for unstable or rare proteins. Though peptide epitope‐imprinted polymers provide potent alternatives to entire protein‐imprinted polymers for protein recognition, the effective epitopes need to be elaborately chosen to ensure the specificity and functionality of the prepared nanoMIPs. For peptide epitopes with specific and spatial conformation, the conformational maintenance during the imprinting process requires strict control over the composition of the polymerization reaction system, limiting the available monomer as well as polymerization methods. Conformational epitopes usually have specific secondary structure, and they are thus easily restricted by the polymeric network of the obtained MIPs. Therefore, template removal should be considered to make the imprinted cavities available for the targeted molecules to bind. More advancements in MIPs for protein recognition are expected to improve nanoMIPs for the active targeting of protein receptors in tumor cells.

### 3.2 Targeting Aberrant Glycans Expressed by Tumor Cells

Glycans are a universal and essential component of living organisms and are abundant in either free form or in covalent complexes with proteins or lipids; they range from a monosaccharide to polysaccharides with thousands of units.^[31]^ Glycans on the cell surface play a vital role in a myriad of biological events, including cellular adhesion and migration, organism development, disease progression, and immunological response.^[32]^ Tumor occurrence and progression are usually accompanied by abnormal changes in glycosylation, making aberrant expression of certain glycan structures potential markers for cancer cells.^[33]^ Commonly observed changes in glycan structures during malignancy encompass abnormal branching of N‐linked glycans (N‐glycans), truncated O‐linked chains, diverse fucosylated and sialylated terminal structures, and alterations in glycosphingolipid expression.[^17a^, ^34^] Unfortunately, ligands that can specifically recognize or bind relevant glycan biomarkers are scarce,^[35]^ which limits the development of glycan‐targeting nanomedicine for cancer therapy.

Taking advantages of molecular imprinting techniques, researchers have developed MIP‐based nanomedicines specifically targeting glycans on the cell surface. To bind oligosaccharides on the cell surface having a glucuronic acid (GlcA) terminal, GlcA‐imprinted polymers were synthesized.^[36]^ The GlcA‐imprinted polymers showed binding capability to phenylglucuronic acid and acetic acid, which have structural similarity with GlcA, while exhibiting less than 1 % cross‐reactivity in binding glucose, galactose, *N*‐acetylglucosamine, and *N*‐acetylgalactosamine. Furthermore, the GlcA‐templated nanoMIP had a high association constant (*K*_a_) with GlcA in dimethyl sulfoxide (7.1×10^3^ M^−1^) due to the noncovalent binding with the functional monomers.^[36b]^ Using carbon nanodots as a core, the GlcA‐imprinted nanoMIP was demonstrated to target human cervical cancer cells by binding hyaluronan.^[36c]^

Initially, only noncovalent forces were used for binding the target monosaccharide in the design of monosaccharide‐binding nanoMIPs.^[36]^ To gain higher affinity to glycans, boronate affinity was introduced later in glycan‐imprinted polymers. Boronate affinity materials have gained increasing attention in the field of glycan recognition since they can undergo reversible covalent binding with *cis*‐diol‐containing compounds controlled by the solution pH.^[37]^ Despite the superior selectivity of boronate affinity materials to *cis*‐diols, they fail to recognize specific *cis*‐diol‐containing compounds. The combination of boronate affinity and molecular imprinting provides an optimal solution for developing targeting reagents to glycans. Boronic acids can be used for facilely anchoring or releasing glycan templates during the imprinting process through control of the solution pH. The imprinted cavities, which preferentially fit the template glycans, and the affinity provided by boronic acids endow the fabricated MIPs with high selectivity and affinity to target glycans. Sellergren et al. designed and fabricated a fluorescent sialic acid (SA)‐imprinted nanoMIP for selectively labeling cell‐surface glycans.^[38]^ The ternary complex imprinting approach including boronate ester, hydrogen bonding, and electrostatic stabilization produced a tailor‐made nanoMIP with exceptional affinity for SA (binding constant *K*=6.6×10^5^ M^−1^ in 2 % water, 5.9×10^3^ M^−1^ in 98 % water). In quantification assays of the expression level of SA, the nanoMIP exhibited a similar staining pattern as lectin but a significantly stronger staining for SA than for lection, indicating a higher affinity to SA. In another study using a facile re‐precipitation of phenylboronic acid modified poly(fluorene‐*alt*‐benzothiadiazole), the fluorescent SA‐imprinted nanoMIP exhibited selective staining for cancer cells overexpressing SA.^[39]^

**Figure 4:**
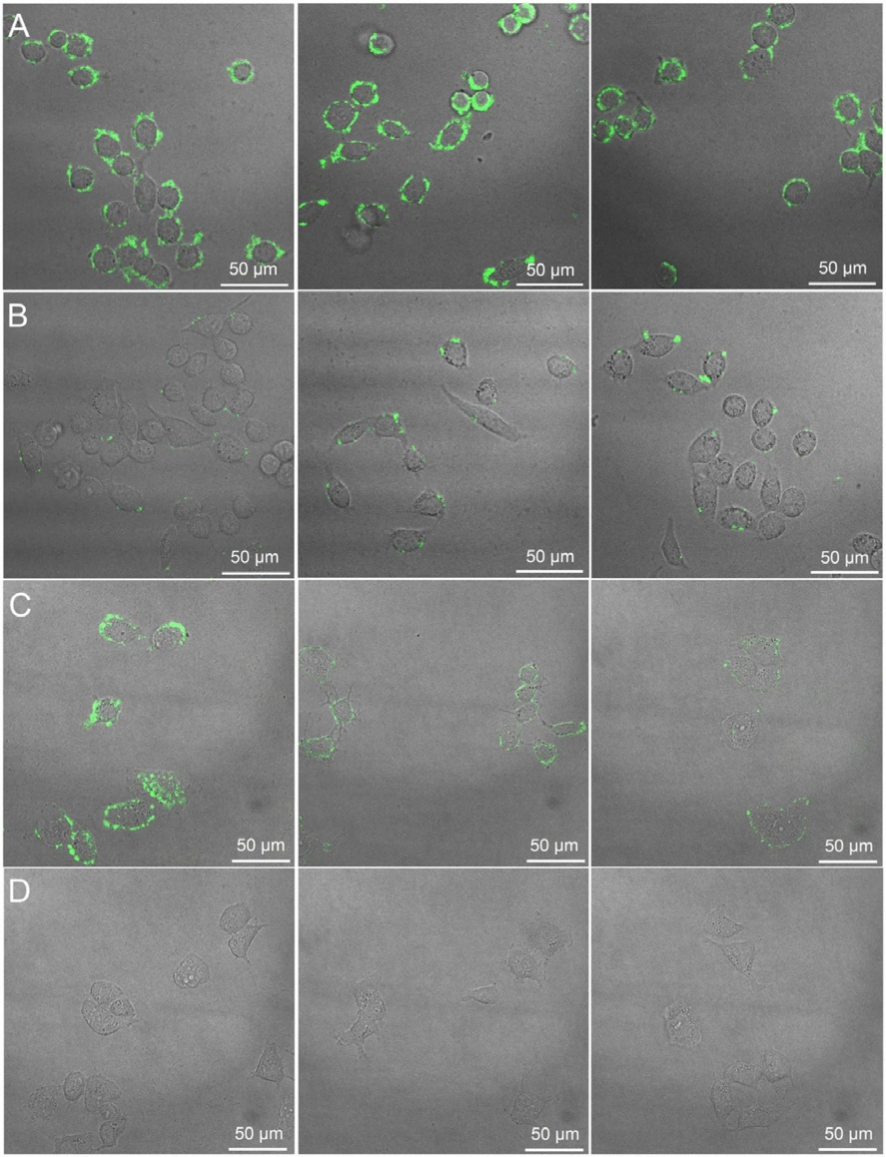
Confocal fluorescence imaging of HepG‐2 cells (A, a liver cancer cell line), L‐02 cells (B, an immortal normal liver cell line), MCF‐7 cells (C, a breast cancer cell line) and MCF‐10A cells (D, a non‐transformed normal breast cell line) after staining with different monosaccharide‐imprinted nanoparticles. Columns from left to right: SA‐, Fuc‐, and Man‐imprinted nanoparticles. The concentration of the nanoparticles was 200 μg mL^−1^.^[40]^ Reprinted with permission. Copyright 2016, Nature Publishing Group.

The monosaccharide‐imprinting strategy for cell targeting was further explored by our group via construction of a general toolbox for specifically recognizing cancer cells by monosaccharide‐imprinted nanoMIPs.^[40]^ SA, fucose (Fuc), and mannose (Man) have been reported to be overexpressed on certain cancer cell surfaces and can serve indicators for some cancers. Based on the boronate affinity oriented surface imprinting approach,^[41]^ nanoMIPs were prepared with different templates including SA, Fuc, and Man. The optimal IF reached up to 8.4, 6.7, and 6.9 for SA, Fuc, and Man, respectively, while the cross‐reactivity toward nontarget monosaccharides was less than 7.1, 26.2, and 22.2 % for SA‐, Man‐, and Fuc‐imprinted nanoMIPs, respectively. The *K*_d_ value of the SA‐imprinted nanoMIP was estimated to be 2.0×10^−4^ M. With high affinity and low cross‐reactivity, the fluorescent monosaccharide‐templated nanoMIPs well reflected the monosaccharide expression level on cell surface (Figure 4). Compared with lectins, the monosaccharide‐binding nanoMIPs provided comparable cancer cell targeting capability but better specificity.^[40]^

Furthermore, the in vivo targeting performance of monosaccharide‐templated nanoMIPs was investigated.^[42]^ SA‐imprinted nanoMIP with gold nanorods (AuNRs) as a core was prepared.^[42a]^ Due to the boronate affinity and imprinted cavities, the SA‐imprinted nanoMIP exhibited excellent specificity and high affinity to SA, leading to enhanced in vivo accumulation at the tumor site compared to NIP nanoparticles. Combined with the plasmonic heating effect of AuNRs, the SA‐binding nanoMIP completely ablated solid tumors in a subcutaneous tumor model. In another study, a hollow double‐layer SA‐imprinted nanoMIP was designed and prepared as a chemotherapeutic for targeted nitric oxide release.^[42b]^ The SA‐imprinted shell made the nanoparticles actively target tumor cells in vitro and in vivo, which greatly improved the antitumor efficacy and reduced the side effects on vital organs in response to chemotherapy. These studies demonstrated the great potential of monosaccharide‐binding nanoMIPs for the active targeting of tumor cells in vivo.

Compared with monosaccharides, the aberrant glycan chains hold more potential as cancer markers with high specificity. NanoMIPs for the recognition of specific glycan chains were developed by our group using the boronate‐affinity controllable oriented surface imprinting approach.[^41^, ^43^] Glycans digested from the target glycoprotein were immobilized onto the boronic acid functionalized nanoparticles through boronate‐affinity interactions and served as imprinting templates. Alternatively, the target glycoproteins could be directly immobilized on the boronic acid functionalized nanoparticles and then digested by peptidase, leaving the glycan peptides as the imprinting templates. With tetraethyl orthosilicate as the monomer, the thickness of the imprinting polymer layer could be controlled to appropriately cover the template, resulting in cavities specific to the template glycans as well as to the intact glycoprotein or glycopeptide containing the template glycan. Using the imprinting approach, HER2‐glycan‐imprinted nanoMIPs were prepared, specifically recognizing HER2‐positive breast cancer cells,^[9e]^ which will be highlighted later in Section 5. The glycan‐imprinting approach provides a wide and efficient avenue for the preparation of nanomedicines targeting glycans.

So far, few active glycan‐targeting cancer nanomedicines have been developed owing to the shortage of glycan‐specific ligands. However, more and more studies support the notion that the aberrant expression of glycans by malignant cells provides a great opportunity to explore glycans as specific targets for cancer cells.[^17a^, ^44^] Regarding the targeting reagents to glycans, MIPs, especially boronate‐affinity‐based MIPs, exhibit significant advantages over traditional biological ligands such as lectins and antibodies with respect to specificity, affinity, stability, and accessibility. Therefore, glycan‐imprinted polymers will become a potent platform for constructing nanomedicines targeting glycans.

## 4 Controlled Release of Antitumor Drugs

Chemotherapy is the mainstay of cancer therapy. Controlling drug release to improve treatment efficacy has been a hot topic in the field. MIPs have been constructed in various devices (e.g. bulk hydrogel, patches, coatings) for ocular, dermal, intravenous, and oral drug delivery.^[45]^ The molecular imprinting technology elicits new polymer formats and shifts the material size to the nanoscale, providing a versatile tool for cancer nanomedicines with controlled drug release. For systemically administered cancer nanomedicines, premature drug release during blood circulation would reduce the amount of the drug arriving at tumors and also cause side toxicity.[^3b^, ^46^] Upon reaching the target site, the nanomedicines are expected to fully release the loaded drugs to achieve an effective therapeutic concentration. In addition to reaching the correct site, the drug delivery system must also sustain an effective drug concentration in tumors to maximize therapeutic efficacy. Aiming at these release kinetics, current nanoMIPs for controlled antitumor drug release emphasize prolonged release and stimuli‐responsive release.

Usually, antitumor drugs serve as template molecules for imprinting. The imprinted cavities and functional reactive residues left after template removal are utilized for drug loading. For drug release, the drug molecules must escape from multiple noncovalent or covalent interactions and diffuse from cavities. During the release of drug molecules from MIPs, the high affinity of the MIPs to the drug molecules results in a more controlled release kinetics.^[47]^ This mechanism has been utilized for the prolonged release of antitumor drugs. One example is a carbazole derivative released from a magnetic nanoMIP prepared with methacrylic acid as the functional monomer and 1,4‐dimethyl‐6‐hydroxy‐9*H*‐carbazole as the template molecule for controlled antitumor drug delivery.^[48]^ Compared to the NIP which displayed total drug release in about 6 h, the nanoMIP prolonged the drug release for more than 48 h. Similar results were also reported by Neda et al., where a 5‐fluourouracil‐templated nanoMIP exhibited a sustained release for more than 96 h, four times longer than the NIP counterpart.^[49]^

Noncovalent forces (including hydrogen bonding, van der Waals forces, and hydrophobic interactions) are generally responsible for the affinity of MIPs to templates. The sensitivity of some interactions to the external environmental pH and temperature renders stimuli‐responsive release kinetics from nanoMIPs,^[47]^ which is favorable for the stimuli‐responsive release of antitumor drugs. Hydrogen bonding is the most commonly employed noncovalent force for template anchoring as well as for drug loading in MIPs. Anti‐neoplastic agents including paclitaxel, 5‐fluorouracil, and DOX have been loaded in their corresponding nanoMIPs, where hydrogen bonding provided the main binding force.[^27b^, ^28^, ^50^] Fast drug release was observed under acid conditions because the disruption of hydrogen bonding weakened the affinity of the imprinted cavities to the drug molecules. Electrostatic interactions were also utilized for stimuli‐responsive release. In work by Peng, hollow microcapsules with DOX‐imprinted shells were designed and prepared to encapsulate and deliver DOX.^[51]^ The imprinted cavities could be blocked by DOX molecules due to electrostatic interactions between amino group of DOX and carboxyl groups of the MIP. The interaction could be weakened at low pH, resulting in a pH‐responsive release. The hollow microcapsules exhibited a sustained release for more than 168 h at pH 6.5, while a burst release delivered 75 % of the loaded drugs in 24 h at pH 5.0. The pH‐sensitive release kinetics contribute to overall drug stability during systematic circulation in a neutral pH environment and rapid release in the acidic environment of the tumor, which is beneficial in cancer therapy. In addition, temperature‐induced fast release was also observed, where the binding force was affected by temperature change.^[52]^

Apart from noncovalent forces, covalent forces were also used by nanoMIPs for stimuli‐responsive release. A functional monomer containing boronic acid derivative was used to prepare a nanoMIP.^[27a]^ Through the pH‐sensitive interaction between the boronic acid group and *cis*‐diols, the prepared nanoMIP effectively loaded *cis*‐diol‐containing bleomycin under alkaline conditions, while quickly and effectively releasing the loaded drug in an acidic environment.^[27a]^

Though imprinted cavities and reactive forces endow molecule‐imprinted nanoMIPs with the controlled release of antitumor drugs, ample room remains for further improvement, with critical barriers that need to be addressed. Almost all nanoMIPs suffer from the initial burst release of their cargo caused by nonspecific absorption on the surface of nanoparticles, a result of the inherent high ratio of surface to volume. To date, the controlled release of antitumor drugs in vivo has not been completely investigated, though several nanoMIPs have been used for antitumor drug delivery in vivo. In addition to the small‐molecule antitumor drugs, the potential of nanoMIPs for the delivery of antitumor macromolecules needs to be explored in the future.

## 5 Antitumor Efficacy

Nanomaterials are generally employed as vehicles to deliver therapeutics to tumor sites in cancer nanomedicine. NanoMIPs, however, not only provide drug delivery platforms but have also been functionally used as therapeutic antagonists to target biologically functional molecules in cancer. With the increasing understanding of tumor occurrence, progression, invasion, and metastasis, considerable tumor‐related signaling pathways have been identified. Activating or blocking the interaction between ligands and receptors can effectively induce apoptosis of tumor cells.^[53]^ The biomimetic recognition of MIPs has been utilized in the design of nanoMIPs for cancer therapy without loading extra drugs.

Catalase (CAT)‐imprinted fibrous SiO_2_ nanoparticles were prepared as a novel nanotrapper for CAT to trigger tumor cell apoptosis.^[54]^ The prepared CAT‐templated nanoMIP efficiently trapped CAT and inhibited its catalytic activity of decomposing hydrogen peroxide (H_2_O_2_). When internalized by tumor cells, the CAT‐imprinted nanoMIP selectively recognized and captured CAT molecules, inducing intracellular accumulation of H_2_O_2_, one of the major reactive oxygen species (ROS). The high ROS level naturally triggered apoptosis of most tumor cells.^[54]^ A similar example was reported by Tang et al.^[55]^ Testosterone (TSTO)‐imprinted nanoMIPs were developed to block the TSTO‐androgen receptor (AR) pathway. These nanoMIPs specifically absorbed intracellular testosterone and then inhibited the cascade of TSTO‐AR pathway related functions as well as the growth of androgen‐dependent prostate cancer cells through suppression of the cell cycle progression.

**Figure 5:**
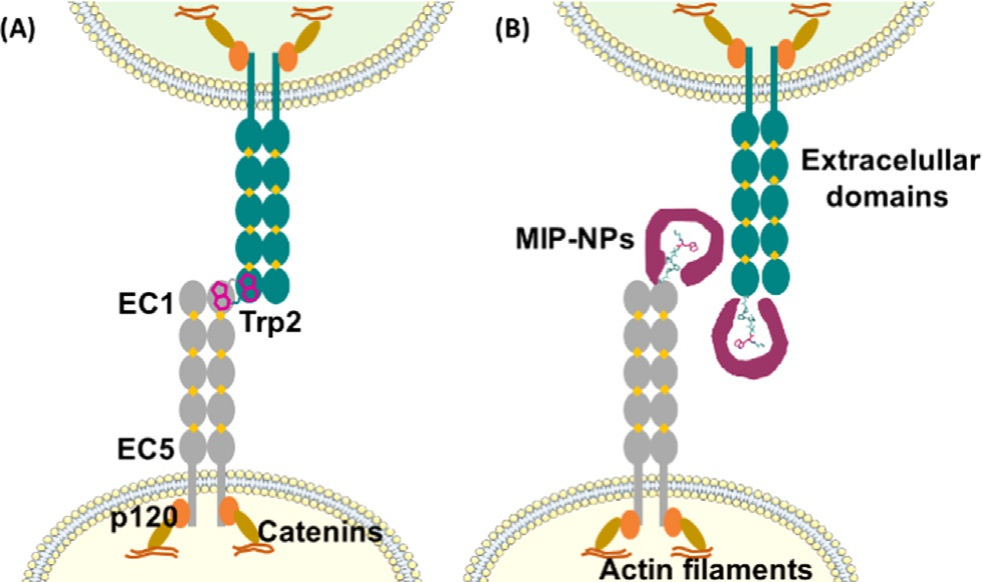
Strand‐intercalation model of adhesive binding by classical type I cadherins. The five ectodomains are represented as gray and green ovals, the different colors symbolize cadherins from opposite cell surfaces. Ca^2+^ ions are shown as yellow rhombi. A) Adhesive binding occurs via N‐terminal strand dimerization. The dimerization involves the insertion of Trp2 (fuchsia) from one cadherin into the hydrophobic pocket located in the EC1 of the partner cadherin. B) Molecularly imprinted polymer nanoparticles (MIP‐NPs) (magenta) targeting an N‐terminal epitope of cadherin, block Trp2 and abrogate cell–cell adhesion.^[9f]^ Reprinted with permission. Copyright 2019, Wiley‐VCH.

In addition to intracellular signal pathways, intercellular interactions could also be regulated by nanoMIPs. Cadherin plays a crucial role in mediating cell–cell adhesion. The N‐terminal peptide epitope of cadherin was used as a template to prepare a nanoMIP by solid‐phase synthesis.^[9f]^ The nanoMIP specifically bound to cadherin on the cell surface, disrupting cell–cell adhesion (Figure 5). Furthermore, the nanoMIP has been shown to disrupt preformed tumor spheroids and inhibit cancer cell invasiveness of HeLa cells in vitro, exhibiting biological activity of modulating cellular function.

**Figure 6:**
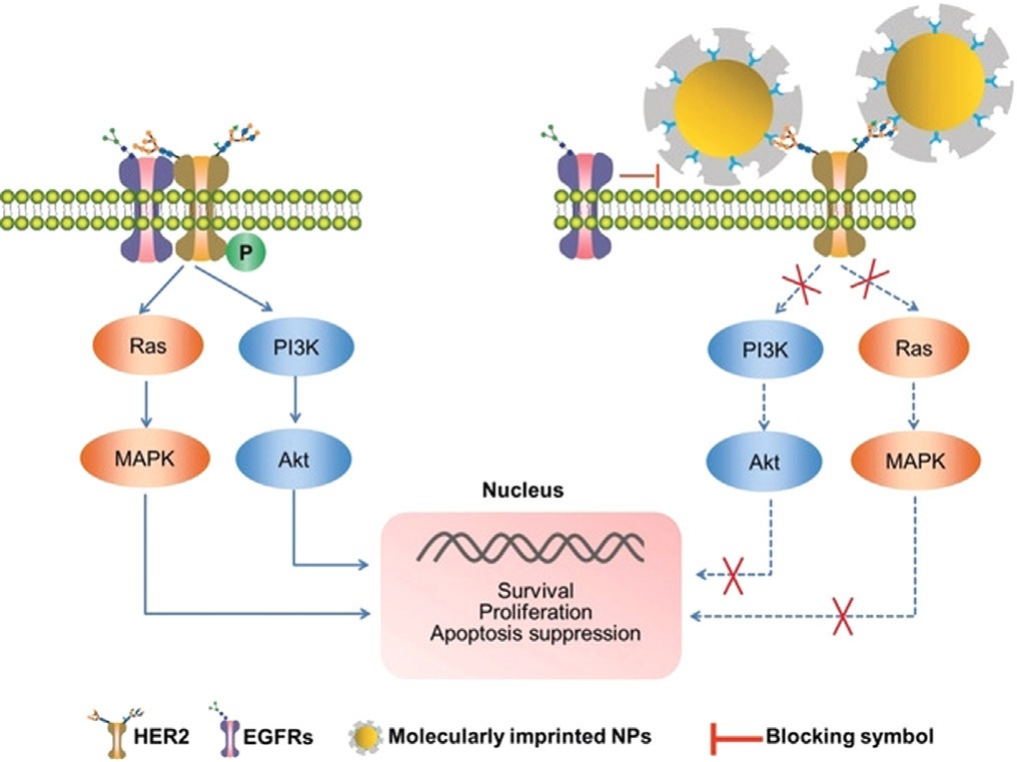
Illustration of the principle of blocking the HER2 signaling pathway via HER2‐glycan‐imprinted nanoparticles.^[9e]^ Reprinted with permission. Copyright 2019, Wiley‐VCH.

The spatial organization of cell‐surface receptors plays an important role in controlling cellular signaling cascades.^[56]^ Controlling the nanoscale distribution of cell binding ligands has been shown to regulate cell behavior, and these can serve as a target for cancer therapy.^[57]^ Heterodimerization of HER2 with other EGFRs at the extracellular subdomain induces phosphorylation of tyrosine residues within the intracellular tyrosine‐kinase domain and triggers several downstream signaling events, resulting in cell proliferation, survival, migration, angiogenesis, and metastasis.^[58]^ Preventing the dimerization of HER2 with EGFRs by monoclonal antibodies provides an effective treatment for HER2‐positive breast cancer.^[59]^ As an alternative to antibodies, the HER2‐glycans‐templated nanoMIP was employed to bind HER2 glycans. This will suppress the dimerization of HER2 with other HER family members to finally block the downstream signaling pathways and inhibit HER2‐positive breast cancer growth (Figure 6).^[9e]^ The nanoMIP specifically inhibited HER2 phosphorylation and proliferation of HER2‐positive tumor cells in a dose‐dependent fashion. The antitumor efficiency was validated in nude mice implanted with human HER2‐positive breast cancer cells.^[9e]^ Without the loading of additional therapeutic agents, the HER2‐binding nanoMIP significantly inhibited tumor growth without showing obvious biological toxicity compared to control groups.^[9e]^

By specifically recognizing and capturing pivotal biomolecules, nanoMIPs could effectively block signaling pathways to directly or indirectly induce antitumor effects. Different from classical nanomaterials serving as vehicles for the targeted delivery of therapeutics, nanoMIPs themselves can exert therapeutic efficacy in a biomimetic and molecularly selective way without the further loading of therapeutic agents. This paradigm is similar to some antibody therapeutics. However, compared with antibodies, nanoMIPs have lower immunogenicity, higher stability, and lower toxicity and side effects. Additionally, nanoMIPs readily penetrate the cell membrane by cell endocytosis to regulate intracellular signals. Although the use of nanoMIPs as cancer therapeutic agents is still at a very early stage, nanoMIPs as drug‐free therapeutics have strong potential for cancer therapy.

## 6 Dual Functions

Owing to the versatile functions of imprinted cavities, a single nanoMIP system can be used to integrate different functions for more effective cancer therapy. The most widely studied dual‐functional nanoMIPs are capable of simultaneously combining target molecules and antitumor drugs as co‐templates. The imprinted cavities provide specific recognition sites as well as drug loading sites. The constructed nanoMIPs could specifically deliver antitumor drugs to tumor cells with controlled kinetics. In line with this concept, several dual templates of nanoMIPs have been constructed and validated to specifically deliver cytotoxic drugs to tumors cells over‐expressing a given receptor, leading to a more profound tumor cell toxicity compared to that of NIP nanoparticles or free drugs. This was attributed to the specific recognition of the nanoMIPs as well as controlled drug release.[^9b^, ^27^, ^28^, ^30^] Furthermore, the in vivo antitumor efficacy was explored in mouse tumor models, demonstrating dual‐functional nanoMIPs as an efficient platform for tumor‐targeted therapy.[^27a^, ^28^, ^30^]

To further advance cancer therapy, it is expected that more dual‐functional and even multi‐functional nanoMIPs will be developed in the near future. For example, to eliminate the insufficient efficacy of single targeting, dual‐targeting nanoMIPs can be constructed to improve tumor cell selectivity and uptake. To reduce drug resistance, combined drug delivery can be designed by using dual‐ or multi‐templated nanoMIPs.

## 7 Summary and Outlook

In this minireview, we have surveyed and highlighted recent advances in the rational design of MIP‐based nanomedicines for cancer therapy. As a polymer material, MIPs feature unique molecular recognition capability with high affinity and specificity. Importantly, MIPs have excellent stability, easy preparation, and low cost compared to traditional bioligands. Given these merits, MIP‐based nanomedicines have exhibited remarkable performance in systemic circulation, specific targeting, and controlled drug release when serving as carriers. Of note, nanoMIPs themselves can also serve as therapeutics by regulating cell signal pathways without loading any drug. This approach holds great potential to provide alternatives to classical chemical and biochemical drugs. Furthermore, nanoMIPs can integrate multiple functions into a single system, presenting a powerful and versatile platform for synergistic therapeutics. This is considered to be a more effective strategy to achieve complete remission and even cures for patients with cancer.

The area of MIP‐based nanomedicines remains in its infancy with many exciting but unexplored areas. In the past, most MIP‐based nanomedicines aimed to address only one issue. However, the successful application of nanomedicine must overcome a cascade of biological barriers. To achieve optimal performance for cancer therapy, a more exquisite and comprehensive design of MIP‐based nanomedicines is critical. For further clinical translation of MIP‐based nanomedicines, the biocompatibility and biodegradability of the nanoMIPs as well as appropriate animal models for in vivo experiments must also be considered and investigated.

Although they faced many challenges at the initial stage, nanoMIPs have opened a new research avenue and exhibit great potentials in cancer nanomedicine. With the progress in the molecular imprinting technology, nanoMIPs and their superior properties will significantly advance cancer therapy.

## Conflict of interest

The authors declare no conflict of interest.

## Biographical Information

*Shuxin Xu obtained her PhD degree from Tianjin University (China) in 2017. She then conducted postdoctoral research at the Suzhou Institute of Biomedical Engineering and Technology. Since 2019, she has been a research associate at Nanjing University. Her current major research interest is biomedical nanomaterials and cancer nanomedicine*.

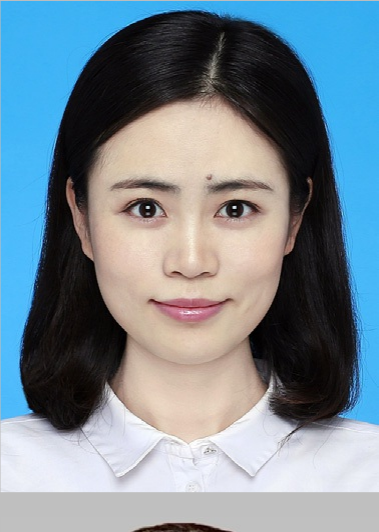

## Biographical Information

*Lisheng Wang obtained his PhD from the Faculty of Medicine, University of Sydney (Australia) in 1999. He then performed postdoctoral studies at Harvard Medical School (USA) and Robert Research Institute (Canada). He was appointed as an assistant professor in 2005 at the Faculty of Medicine, University of Ottawa, Canada and promoted to associate professor and full professor thereafter. One of his lab's research interests is the use of nanomedicines in breast cancer therapy*.

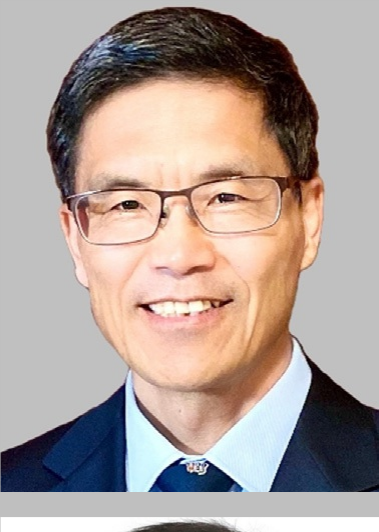

## Biographical Information

*Zhen Liu obtained his PhD from Dalian Institute of Chemical Physics in 1998. Then he performed postdoctoral studies at the University of Hyogo (Japan) and the University of Waterloo, (Canada). He was appointed as a full professor at Nanjing University in 2005 and promoted to Distinguished Professor in 2014. He was awarded the 2020 Advances in Measurement Science Lectureship Award by American Chemical Society. His research interests include molecular imprinting and cancer nanotherapy*.

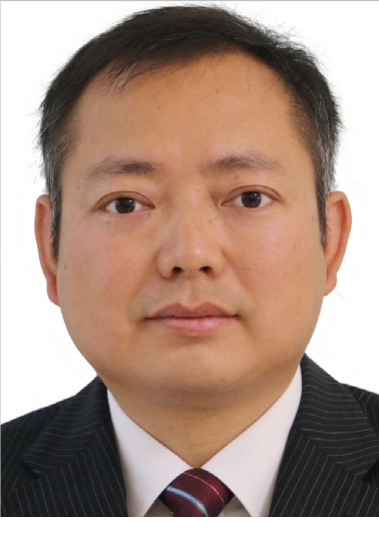
